# Catalysts as Sensors—A Promising Novel Approach in Automotive Exhaust Gas Aftertreatment

**DOI:** 10.3390/s100706773

**Published:** 2010-07-13

**Authors:** Ralf Moos

**Affiliations:** University of Bayreuth, Bayreuth Engine Research Center, Bayreuth, Germany; E-Mail: Functional.Materials@Uni-Bayreuth.de; Tel.: +49-921-55-7401

**Keywords:** on-board diagnosis (OBD), lambda probe, three-way catalyst (TWC), microwave, ammonia SCR, lean NO_x_ trap (LNT)

## Abstract

Sensors that detect directly and *in situ* the status of automotive exhaust gas catalysts by monitoring the electrical properties of the catalyst coating itself are overviewed. Examples included in this review are the *in-situ* determination of the electrical impedance of three-way catalysts based on ceria-zirconia solutions and of lean NO_x_ traps of earth-alkaline based coatings, as well as approaches to determine the ammonia loading in Fe-SCR-zeolites with electrical ac measurements. Even more sophisticated approaches based on interactions with electromagnetic waves are also reviewed. For that purpose, metallic stick-like antennas are inserted into the exhaust pipe. The catalyst properties are measured in a contactless manner, directly indicating the catalyst status. The radio frequency probes gauge the oxygen loading degree of three-way catalysts, the NO_x_-loading of lean NO_x_ traps, and the soot loading of Diesel particulate filters.

## 1. Introduction

In the past years, many efforts were being made to develop novel automotive exhaust gas sensors that are sensitive, selective, have long-term stability, and are cost effective (see for instance Refs. [1–6] and the literature quoted therein). However, as of today, only zirconia-based sensors like the binary lambda probe, the universal exhaust gas oxygen sensor (also known as UEGO sensor or linear lambda probe), and the amperometric NO_x_-sensor have been serialized. Besides direct engine control, all these selective gas sensors are applied in the exhaust to detect the status of the catalyst *indirectly* with the help of models. Depending on the catalyst type, status in this respect may mean:

○ Current oxygen loading of three-way catalysts (TWC)
○ Current NO_x_-loading of lean NO_x_ traps (LNT)
○ Current NH_3_-loading of ammonia-SCR catalysts (SCR)
○ Soot loading of Diesel particulate filters (DPF)
○ Conversion efficiency
○ Sulfur poisoning
○ And others.

Recently, a novel approach emerged. The catalyst status is detected *directly* by monitoring the electrical properties of the catalyst coating itself. This article reviews several attempts in this field. Overviewed examples include:

○ *In-situ* monitoring of the impedance of ceria-zirconia based TWCs to determine their degree of oxygen loading,
○ *In-situ* measurement of the impedance of earth-alkaline oxide-based LNT coating materials to detect the status of an LNT with respect to its NO_x_-loading, its status of regeneration, its degree of sulfurization, and its thermal aging,
○ Approaches to determine the ammonia loading in Fe-SCR-zeolites with electrical ac measurements.

Even more sophisticated and extremely promising are very recent microwave-based approaches. Here, radio frequency antennas in the form of simple metallic sticks are inserted into the exhaust pipe and the reflection parameters or the transmission parameters are determined. Such a system measures the catalyst properties in a *contactless* manner indicating directly the catalyst status.

It will be shown that these measurements allow to detect:

○ The oxygen loading degree of a TWC
○ The NO_x_-loading of an LNT and
○ The soot loading of a DPF.

## 2. Background

Increasing fuel costs combined with the pressure on the automotive industry to reduce CO_2_ emissions has lead to booming market shares for Diesel passenger cars. Since Diesel engines are operated leanly, NO_x_ removal with conventional three-way catalysts is not possible, e.g., [6,7]. Therefore, novel exhaust gas aftertreatment concepts are required.

The ammonia SCR process has been adapted for automotive requirements. For heavy duty vehicles, systems are already in serial application and they have recently also been serialized for passenger cars [8]. In NH_3_-SCR systems, an ammonia forming substance, e.g., a urea water solution, is injected into the exhaust. In the SCR catalyst, NH_3_ is formed, serving as a selective reduction agent for NO_x_. According to the reaction mechanism, NH_3_ is initially adsorbed (stored) in the SCR-catalyst. Especially at low temperatures, NO_x_ reduction (conversion rate) is strongly dependent on the amount of stored NH_3_ [9,10].

In lean NO_x_ traps (LNT), NO_x_ is adsorbed and stored in the form of nitrates during a lean phase. Nitrate reduction occurs in a subsequent short rich phase, just before the NO_x_ storage capacity of the LNT is exhausted and it begins to let pass NO_x_ [11]. The fact that due to sulfur oxide in the exhaust, sulfates form and prevent further NO_x_ storage is a critical issue. Then, an energy-consuming desulfurization phase is required, which should be initiated as seldom as possible [12]. For both novel NO_x_ removal technologies, NH_3_-SCR and LNT, novel exhaust gas sensors that provide information on the status of the catalyst (amount of stored NO_x_, degree of sulfate-poisoning, amount of stored NH_3_) to supplement the well-known and mature lambda probe would be helpful for catalyst control.

Due to the strongly reduced particulate matter limits, Diesel particulate filters have been serialized. DPFs must be regenerated on a regular basis to burn off the soot adsorbed in the course of time [13]. As the regeneration processes consume fuel, the number of regenerations must be kept to a minimum. Hence, a detailed knowledge of the actual soot loading of the filter is required.

The exhausts of most gasoline-run internal combustion engines are cleaned by three-way catalyst converters [6,7]. A stoichiometric air-to-fuel ratio (λ = 1) is required for best conversion of limited exhaust components, like NO_x_, hydrocarbons, or CO. For this purpose, lambda probes are installed upstream and downstream of each catalyst to determine the current air-to-fuel ratio in the gas phase [1,2]. In order to buffer lean-to-rich fluctuations, the TWC washcoat (catalytically active coating of the honeycomb-like substrate) contains large amounts of doped ceria-zirconia solutions as an oxygen storage component, making use of the two different oxidation states of cerium at exhaust gas temperatures. The total oxygen storage capacity (OSC) is directly related to the amount of ceria present [14]. With λ measurements up- and downstream of a TWC, its degree of oxidation is calculated by model-based approaches. This indirect characterization of the catalyst’s oxygen loading degree is state-of-the-art.

To summarize this section, it becomes obvious that almost all exhaust gas aftertreatment devices store gases or particles. It would be helpful to determine directly the loading status with catalyst status sensors. In the past few years, a novel approach emerged. It has been investigated, whether and in which cases the catalyst status can be gauged directly by monitoring the electrical properties of the catalyst coating itself.

## 3. Impedance-Based Direct Catalyst Diagnosis

### 3.1. Three-Way Catalyst (TWC)

**Figure 1.**
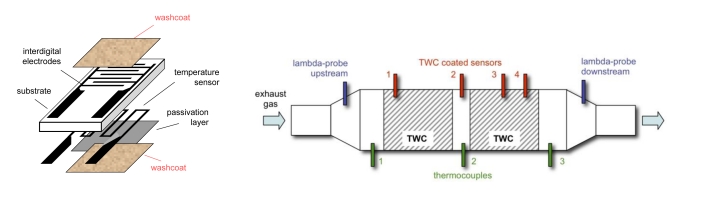
Schematic sensor element setup (left). Sensor positions in the catalyst (right). The numbers 1, 2, 3, and 4 denote the location of sensor elements. Results obtained from these sensors are shown in Figure 2. Modified after [17].

According to well-known defect chemistry [15], the washcoat changes its electrical conductivity in dependence of the oxidation state of ceria. This effect has been investigated for conductometric exhaust gas oxygen sensors [16], but it can also be used to directly determine the oxygen loading status of a TWC [17]. For that purpose, a TWC washcoat is applied onto interdigitated electrodes (IDE), which are inserted into the catalyst. A typical sensor element setup is depicted in Figure 1 (left). Several of these sensor elements are arranged in a catalyst housing along the flow axis (Figure 1, right, numbers 1–4) to spatially resolve the local loading degree in the catalyst. The sensors are heated by the exhaust, *i.e.*, their operation temperature is the temperature of the exhaust gas.

In other words, since the oxygen partial pressure changes by 10 to 20 decades between lean and rich [1], a strongly changing resistance can be expected if the sensor film (=the washcoat) varies its oxygen loading degree from fully loaded to completely depleted.

Reiß *et al.* [17] conducted lean-rich switches both in synthetic exhausts and in dynamometer test benches. It was clearly shown how an oxygen loading front moves through the catalyst. Furthermore, by normalizing and summing up the signals over the length of the catalyst, an overall oxygen loading could be determined, which agrees very well with the values obtained from the oxygen balance determined by analyzing the exhaust upstream and downstream of the TWC [18].

**Figure 2.**
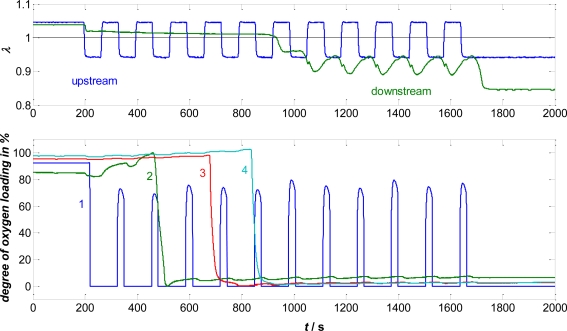
Oxygen loading experiment with the setup of Figure 1. Air-to-fuel ratio, measured by lambda probes (top). Sensor conductivity expressed in terms of % oxygen loading (bottom). Modified and recalculated using data from [17].

A very interesting experiment is shown in Figure 2. The gas composition was switched from lean to rich. After a lean period lasting until *t* ≈ 200 s, an alternation between rich and lean gas (each for 1 min, net slightly rich) was conducted. The upstream lambda probe mirrors these changes clearly. The second λ-signal remains constant at about λ ≈ 1 during the switches, until at *t* ≈ 900 s λ-oscillations occur downstream of the TWC.

The washcoat conductivity, here expressed as an oxygen loading degree (details in [18]), measured with the first sensor follows the different gas compositions albeit slightly time delayed. The remaining sensor devices do not show a significant oscillation, but rather a distinct switch from loaded to unloaded. This behavior can be explained well if one assumes an oxidation (or reduction) front moving through the catalyst in the gas flow direction. Since diffusion into the coating and reaction of the gas with noble metals and ceria can be considered to be much faster than the oxygen transport along the flow axis [14], the first few centimeters of the TWC buffer most of the fast lean-rich switches. Hence, not λ-alterations but only an overall oxygen depletion zone moves through the catalyst, as clearly shown by the direct oxygen loading sensor.

### 3.2. Lean NO_x_ Trap (LNT)

A similar approach has been intensively investigated for lean NO_x_ traps. Here, the situation is more complex, because the washcoat has not only the functionality to store oxygen but mainly stores NO_x_ in the form of nitrates. Details of this principle can be found in [6,11,19]. Similar to Figure 1, an IDE is applied on an electrically insulating ceramic plate. It is coated with the identical coating of the ceramic LNT monolith. The sensor is operated at the same temperature as the LNT, e.g., at 300 °C. The sensor impedance is determined at some kHz.

**Figure 3.**
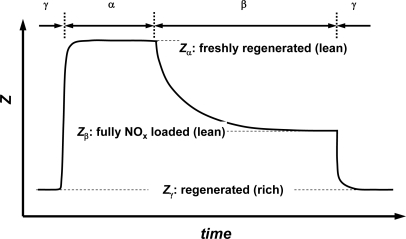
Sketch of the electrical impedance of LNT materials at 350 °C. Simplified after Refs. [20] and [21].

Zimmermann *et al*. initially studied the principal behavior (Figure 3, after [20,21]). Different gas compositions (“α”, “β”, “γ”) are applied to the sensor one after the other. During the rich composition “γ” with λ ≈ 0.8, nitrate reduction occurs. It is followed by a lean composition “α” with λ ≈ 2.1. In contrast to the real engine process, “α” does not contain NO_x_. This is to distinguish in the basic experiment between the effect of NO_x_ storage and oxygen storage.

The NO_x_ storage phase does not begin until “β” (a composition like “α” but with NO) is applied. The electrical impedance, *Z*, takes three final values. They denote three different states of the LNT: *Z*_γ_ (rich, regenerated), *Z*_α_ (lean and freshly regenerated) and *Z*_β_ (lean and NO_x_ loaded). Let us start our considerations at the lean regenerated point, *Z*_α_. As soon as NO is added to the exhaust, the catalyst coating stores NO_x_ and the impedance decreases as the NO_x_-loading increases until *Z*_β_ reaches the final value for “fully NO_x_ loaded in the lean”. Because LNT and sensor material are identical, it is clear that the catalyst loading degree can be determined directly with the help of the electrical measurement. Of course, the temperature dependence of the electrical impedance has to be corrected [21]. It should be noted, as an aside, that if one uses an LNT material which stores all offered NO_x_, but which does not release NO_x_ under the absence of NO_x_, one can even design an integrating sensor device utilizing this principle [22].

**Figure 4.**
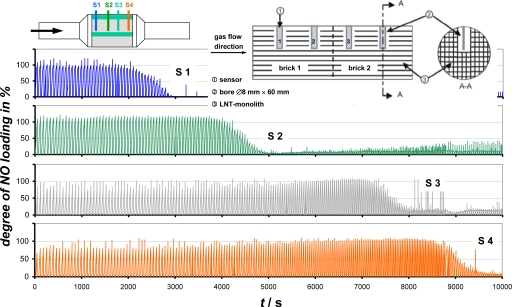
Engine dynamometer test of four sensors representing the status of the LNT coating along the flow axis as indicated. SO_2_ is added to speed up sulfurization. Please note: the increasing spike frequency is a result of the increased regeneration frequency initiated by the engine control to compensate the decreasing NO_x_ storage capability due to sulfur poisoning. From [20] and [21]. Reprinted with permission from SAE paper 2008-01-0447 © 2008 SAE International.

Engine dynamometer tests were conducted as well [20,21,23]. Four sensors, each having the same LNT coating, were inserted in an LNT. The sensor positions are indicated in Figure 4. The canned catalyst consisted of two LNT bricks. Two sensors were inserted in each of them (S1…S4). Sensors and LNT were treated in the same manner. Therefore, the sensor can be regarded as a representative of the catalyst coating at the sensor position. The first sensor represents the first part of the catalyst; the other sensors stand for the behavior along the flow axis. An electronic circuit was built to calculate a “degree of NO-loading” from the impedance values of each sensor based on a previous correlation between the amount of stored NO in the LNT and the sensor impedance. In this experiment, the LNT was operated at an exhaust temperature of 300 °C. SO_2_ was added to the exhaust to sulfur-poison the LNT within a few hours.

The obtained loading signals in Figure 4 clearly show the sulfur poisoning front proceeding through the LNT. During the rich-lean cycles, the loading at S1 varies between 0 and 100%. At the outset, the NO_x_-loading at S3 and S4 never reached 100%. Due to the sulfur poisoning front moving towards the end of the LNT, one sensor after the other loses its storage behavior. With increased sulfur uptake, the NO_x_-storage process occurs in the rear parts of the LNT. It could even be demonstrated how desulfurization occurs [20].

### 3.3. Ammonia-SCR-Catalyst

NO_x_-conversion depends on the degree of ammonia loading of the SCR-catalyst [24]. Especially at low temperatures, a high conversion rate goes along with a well NH_3_-loaded SCR-catalyst. The actual degree of ammonia loading (sometimes also called surface coverage ratio [25]) can be determined by complex models that are based on the total NH_3_ storage capacity of the SCR-coating as a function of the engine operation data in conjunction with a NO_x_-balance using NO_x_ sensors. Direct measurement utilizing an ammonia loading sensor would probably be more accurate. Again, the basic idea is to use a part of the catalyst material itself as a sensor for that [26].

The impedance of a film of catalyst material depends on the amount of stored ammonia. During a short local temperature increase in which ammonia desorbs, the conductivity is measured. The conductivity change can be seen as a measure for the ammonia loading of the catalyst. A similar setup to the TWC oxygen loading sensor as sketched in Figure 1 (left) is used, however the temperature sensor on the lower side is replaced by a heater film.

**Figure 5.**
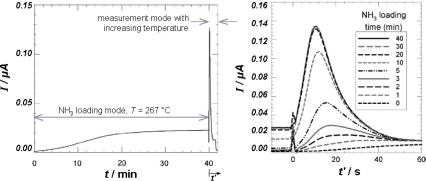
Current, *I*, as a result of an applied ac voltage (left). At *t* = 0, NH_3_ was added to the feed gas and the catalyst film got loaded. After 40 minutes, the temperature was actively increased (measuring mode starts at *t*’ = 0). Course of the current in the measuring mode for different loading times (right). Loading temperature always 267 °C. Modified after [26], reprinted with permission from Elsevier.

Fundamental tests were conducted with a zeolite SCR material in synthetic exhaust with 5% O_2_, 1% H_2_O and N_2_ by Kubinski and Visser [26]. At the beginning, the catalyst coating is free of stored ammonia. During the loading mode at constant temperature, ammonia is added to the feed gas and is stored in the film. During the following measuring mode, the temperature is increased utilizing the heater on the sensor bottom side and stored NH_3_ desorbs and/or gets oxidized. In this case, the measurand is the amplitude of an ac current at an applied constant voltage amplitude. It is proportional to the complex electrical conductance. Figure 5 shows a typical result.

During loading, the current increases since the electrical conductivity of the zeolite increases with ammonia loading, as known from zeolite ammonia sensors [27–29] or zeolite-based *in situ* ammonia diagnostics in a TPD [30]. The final value depends on the NH_3_-concentration of the gas, since the equilibrium amount of stored NH_3_ is a function of the NH_3_-concentration in the exhaust. During the measuring mode (heating, *t*’ > 0), the measured current increases further due to the thermally activated electrical conductivity. With increasing release of the stored ammonia, the current decreases strongly. The signal maximum increases with increasing NH_3_-loading time and reaches a saturation level after a distinct loading time. The loading time until the saturation level is reached depends on temperature and on the NH_3_ concentration of the gas. The area under these curves correlates with the stored amount of ammonia ([26] with data from [10]). The advantage of this procedure is that the catalyst material itself serves as a sensor and shows directly the loading degree - and possibly, at least by plausibility considerations-, aging, and functionality of the SCR system.

## 4. Radio Frequency-Based Contactless Direct Catalyst Diagnosis

### 4.1. Oxygen Loading of a TWC

**Figure 6.**
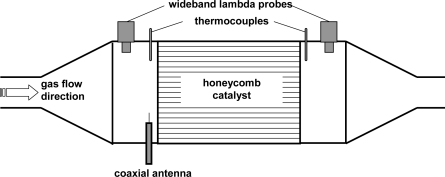
Schematic test setup for verifying the principle of the radio frequency-based catalyst status detection method.

As shown above, the electrical conductivity of washcoat materials depend on the catalyst loading state. In the case of TWC, the conductivity of the washcoat component ceria-zirconia is a function of the oxygen loading [31]. Hence, by utilizing interactions of the catalyst material with microwaves it might be suitable to measure directly and in a contactless manner the catalyst state. A typical test setup is shown in Figure 6.

A radio frequency (rf) signal above the cut-off frequency is applied on the catalyst device by a coaxial antenna. The canning of the ceramic monolith is an electrical conductor. From an electrical standpoint, the setup can be seen as a partially filled circular waveguide. More details of the radio frequency setup are explained in Ref. [32] and first results on engine dynamometers are discussed in [33]. At some distinct frequencies, which depend on the catalyst geometry and on the material permittivity, resonances occur and the so-called input reflection coefficient, *S*_11_, which denotes the ratio of the backscattered and the impinged wave, shows prominent minima. A change in the conductivity of the catalyst coating should have an influence on the *S*_11_-spectra.

**Figure 7.**
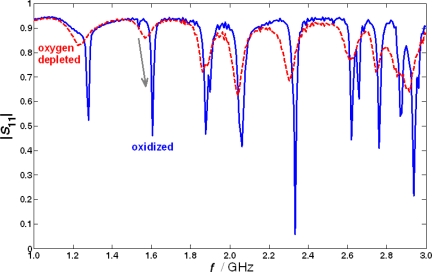
Spectrum of the input reflection coefficient of a TWC when fully oxidized or oxygen-depleted (engine test; *T* ≈ 450 °C, space velocity 60,000 h^−1^, catalyst size approx. ∅118 mm × 127 mm).

This is demonstrated in Figure 7. Again, lean-rich switches were conducted. Very clear differences can be observed between the oxidized (lean) and the oxygen-depleted (rich) state, denoting the two levels of the oxygen buffer. Possible signal characteristics that may serve as measurands are the value of *S*_11_ at the resonance frequency, *f*_res_, or the resonance frequency, *f*_res_, itself. In the following, only the resonance frequency, *f*_res_, is considered.

**Figure 8.**
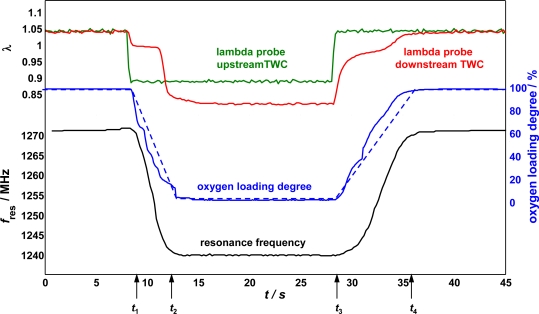
Lambda probe signals up- and downstream of the TWC (green rsp. red), resonance frequency (*f*_res_) during oxygen loading and unloading (black), and measured (blue) and roughly calculated (dotted blue) degree of oxygen loading. Recalculated, partly using data from [18].

It is interesting to see what happens if one observes in detail the transition from lean to rich. For that, the resonance frequency was tracked during lean-rich switches (Figure 8). The output signals of the lambda probe (Figure 8, top, green and red) indicate the engine operating conditions. When the TWC is completely oxygen loaded, the engine is switched to rich combustion (at *t*_1_ ≈ 9 s). After approximately 4 s at *t*_2_, the buffer phase is over, since the oxygen storage capacity of the TWC is exhausted. Then, λ downstream of the TWC becomes rich. The lambda probe downstream of the TWC (red) measures a too low λ value due to its hydrogen cross sensitivity [34]. At *t*_3_ ≈ 28 s, the subsequent oxygen loading phase begins. The TWC stores oxygen until it is fully oxygen-loaded at *t*_4_ ≈ 36 s. Since the deviations from the stoichiometric value is lower by a factor of two, the oxygen loading (*t*_4_ − *t*_3_ = 8 s) takes twice as long as the oxygen release (*t*_2_ − *t*_1_ = 4 s). As soon as the TWC starts to release oxygen, the resonance frequency (black curve) decreases continuously until a constant value is reached. During the subsequent oxygen loading phase, *f*_res_ increases again with a constant slope to a value that indicates the oxidized state. The oxygen loading degree is determined by roughly balancing oxygen up- and downstream of the TWC (blue dotted curve). In addition, it is measured by nine TWC status control sensors as shown in Figures 1 and 2 (blue drawn curve). The sensors are positioned along the flow axis and the averaged degree of oxygen loading of the catalyst is calculated as a result of a weighted summing up of each of the nine sensor signals (details in [18]). The good agreement between resonance frequency and analytically determined oxygen loading proves that the resonance frequency is a suitable measure to determine the oxygen loading degree of a TWC. The influence of CO, H_2_ and H_2_O are investigated as well and are found out to be very low, suggesting that the gas composition dependency of the rf-system is lower than it is for a lambda probe [35]. It will be interesting to evaluate whether the rf-system is also capable to detect catalyst aging. At the moment, it seems possible to establish an alternative control concept that does not need two lambda probes.

### 4.2. NO_x_-loading of LNTs

The conductivity of LNT-coatings varies with its degree of NO_x_-loading as well with the amount of stored oxygen. Therefore, one might expect to determine the status of NO_x_-loading with a similar rf-setup as shown in Section 4.1. In an initial attempt, which is, however, by far not as developed as for TWCs, it has been demonstrated that one can distinguish between oxygen loading and NO_x_-loading [23]. As expected from Section 3.2., the effects of NO_x_-loading are much smaller, but it is assumed that also the degree of sulfur poisoning can be detected. No engine test data exist at the moment.

### 4.3. Soot Loading of DPFs

A novel idea is to monitor the amount of soot deposited on a Diesel particulate filter (DPF) by the radio frequency method. Since soot has a noticeable conductivity, it makes sense to investigate how soot loading affects the resonance spectra.

DPFs are ceramic wall-flow filters with alternate plugged channels. The exhaust is forced through the porous channel walls, in which the particulate matter is trapped. A detailed overview on DPF technology can be found in refs. [13,36,37]. With increased soot loading, the flow resistance increases. When a soot loading of several g/L filter volume is deposited, the particulate filter has to be regenerated to avoid clogging and increasing fuel consumption, *i.e.*, soot has to be oxidized at higher exhaust temperatures. In serial applications, a pressure sensor determines the pressure differences up- and downstream of the DPF. By applying a complex pressure model at specific volumetric flow rates, a determination can be made when the filter requires soot regeneration [6]. At the moment, only soot sensors are under serial development [38–41]. They determine the amount of soot particles in the exhaust flow but not the filter loading. Although it has been shown recently that coke deposits in industrial fixed bed catalysts can be directly and *in-situ* monitored by impedance spectroscopy of a representative catalyst pellet [42], on a soot loading sensor measuring directly the impedance of a DPF has not been reported in the scientific literature.

**Figure 9.**
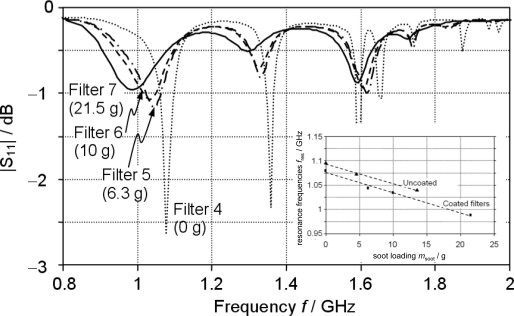
Reflection spectra obtained for DPFs with different soot loadings as indicated. DPF volume: 2.3 liter. Please note: in contrast to Figure 7, |*S*_11_| is given in dB. Slightly modified after [43], reprinted with permission from Institute of Physics and IOP Publishing 2010.

However, a very promising approach has been proposed recently. In a setup similar to Figure 6, soot filters were investigated. It is shown in Figure 9 that a unique relationship between soot loading and the characteristics of the scattering parameters exists [43]. Suitable signal characteristics are resonance frequencies, resonance peak bandwidths, attenuation at resonance and fractional power loss. The inset of Figure 9 depicts the shift of the resonance frequency with soot loading of coated as well as of uncoated DPFs (2.3 liter filter volume). The authors of [43] state that the measurement principle has been validated enough. Further tests should be directed to investigate the influence of disturbing quantities like temperature and agglomeration of unburnt hydrocarbons or of water.

## 5. Conclusions and Outlook

Together with the reviewed results on rf methods for the oxygen loading of three-way catalysts and the NO_x_-loading of lean NO_x_ traps, all major catalyst devices can be monitored in a contactless manner. While for real world applications the rf method might be especially interesting due to its simple and inexpensive setup, the impedance-based approach might lead to a deeper understanding of the catalyst materials and their interplay with the gas phase. In any case, both direct methods provide a novel insight into the state of the catalyst. They might not replace but rather support the commonly used exhaust gas sensors.

Despite the fact that the above-shown data are very promising, it is clear that both measurement techniques—the impedance-based and the rf-based method—are far from serial application in the near future. The influence of disturbing quantities has to be investigated more in detail, as well as the suitability for long-term application in the exhaust has to be demonstrated. The rf-based measuring technique may look charming at a first glance, but electromagnetic compatibility issues have to be considered and suitable sensor electronic prototypes needs to be developed prior one can even think of a serial application. And after all, one has to demonstrate not only the technical merits but also to prove that an rf-based system provides also economic benefits - a must in the automotive industry.
